# Lenalidomide Maintenance with or without Prednisone in Newly Diagnosed Myeloma Patients: A Pooled Analysis

**DOI:** 10.3390/cancers11111735

**Published:** 2019-11-05

**Authors:** Francesca Bonello, Stefano Pulini, Stelvio Ballanti, Massimo Gentile, Stefano Spada, Ombretta Annibali, Paola Omedé, Sonia Ronconi, Clotilde Cangialosi, Luigi Podda, Angelo Palmas, Alessandra Malfitano, Giulia Rivoli, Angelo Belotti, Fabrizio Ciambelli, Iolanda Donatella Vincelli, Anna Maria Cafro, Vanessa Innao, Antonio Palumbo, Pieter Sonneveld, Andrew Spencer, Roman Hájek, Mario Boccadoro, Francesca Gay

**Affiliations:** 1 Myeloma Unit, Division of Hematology, University of Torino, Azienda Ospedaliero-Universitaria Città della Salute e della Scienza di Torino, via Genova 3, 10126 Torino, Italy; francesca.bonello@edu.unito.it (F.B.); stefano.spada@fonesa.it (S.S.); paolaomede@yahoo.com (P.O.); amalfitano@cittadellasalute.to.it (A.M.); Antonio.Palumbo@takeda.com (A.P.); mario.boccadoro@unito.it (M.B.); 2 U.O. Ematologia Clinica, Dipartimento di Ematologia, Ospedale Civile “Spirito Santo”, 65124 Pescara, Italy; stefanopulini0@gmail.com; 3 Ematologia con TMO, Ospedale Santa Maria della Misericordia, 06129 Perugia, Italy; stelvioballanti@alice.it; 4 UOC di Ematologia, AO Cosenza, 87100 Cosenza, Italy; massimogentile@virgilio.it; 5 Unit of Hematology, Stem Cell Transplantation, University Campus Bio Medico, 00128 Rome, Italy; O.Annibali@unicampus.it; 6 Istituto Scientifico Romagnolo per lo Studio e la Cura dei Tumori (IRST) IRCCS, 47014 Meldola, Italy; sonia.ronconi@irst.emr.it; 7 Divisione Ematologia UTMO, Azienda Ospedaliera Villa Sofia Cervello, 90146 Palermo, Italy; clotildecangialosi@hotmail.com; 8 Ematologia, Azienda Ospedaliero-Universitaria Sassari, 07100 Sassari, Italy; podda@uniss.it; 9 Ematologia, Ospedale S. Francesco, 08100 Nuoro, ATS Sardegna, Italy; angelo.palmas@gmail.com; 10 Haematology Clinic, Department of Internal Medicine (DiMI), University of Genoa, Policlinico San Martino, IRCCS per l’Oncologia, 16132 Genova, Italy; giulia.riv@gmail.com; 11 Division of Hematology—ASST Spedali Civili di Brescia, 25123 Brescia, Italy; ange.belotti@gmail.com; 12 Sc Ematologia, ASST Valle Olona, 21052 Busto Arsizio, Italy; ciambelli@libero.it; 13 Divisione di Ematologia, Grande Ospedale Metropolitano Bianchi-Melacrino-Morelli, 89124 Reggio Calabria, Italy; donatella.vincelli@gmail.com; 14 Department of Oncology and Hematology, Niguarda Ca’Granda Hospital, 20162 Milano, Italy; annamaria.cafro@ospedaleniguarda.it; 15 Division of Hematology, Department of Human Pathology and the Adult Developmental, Faculty of Medicine, Messina University, 98122 Messina, Italy; vinnao@unime.it; 16 Department of Hematology, Erasmus MC Cancer Institute, 3015 GD Rotterdam, The Netherlands; p.sonneveld@erasmusmc.nl; 17 Department of Haematology, Alfred Health-Monash University, VIC 3004 Melbourne, Australia; aspencer@netspace.net.au; 18 Department of Haematooncology, University Hospital Ostrava, 70852 Ostrava, Czech Republic; roman.hajek@fno.cz; 19 Faculty of Medicine, University of Ostrava, 70300 Ostrava, Czech Republic

**Keywords:** multiple myeloma, newly diagnosed, maintenance, lenalidomide, prednisone

## Abstract

We conducted a pooled analysis of two phase III trials, RV-MM-EMN-441 and EMN01, to compare maintenance with lenalidomide-prednisone vs. lenalidomide in newly diagnosed transplant-eligible and -ineligible myeloma patients. Primary endpoints were progression-free survival, progression-free survival 2 and overall survival with both regimens. A secondary aim was to evaluate the impact of duration of maintenance on overall survival and on outcome after relapse. A total of 625 patients (lenalidomide-prednisone arm, *n* = 315; lenalidomide arm, *n* = 310) were analyzed. The median follow-up was 58 months. Median progression-free survival (25 vs. 19 months; *p* = 0.08), progression-free survival 2 (56 vs. 49 months; *p* = 0.9) and overall survival (73 months vs. NR; *p* = 0.08) were not significantly different between the two arms. Toxicity profiles of lenalidomide-prednisone and lenalidomide were similar, with the exception of neutropenia that was higher in the lenalidomide arm (grade ≥ 3: 9% vs. 19%, *p* < 0.001), without an increase in the rate of infections. Overall survival (median NR vs. 49 months, *p* < 0.001), progression-free survival from relapse (median 35 vs. 24 months, *p* = 0.004) and overall survival from relapse (median not reached vs. 41 months, *p* = 0.002) were significantly longer in patients continuing maintenance for ≥2 years. We showed that the addition of prednisone at 25 or 50 mg every other day (eod) to lenalidomide maintenance did not induce any significant advantage.

## 1. Introduction

Multiple myeloma (MM) accounts for approximatively 1.8% of all neoplasia and 13% of all hematologic malignancies [1]. An improvement in overall survival (OS) has been reported in recent years, with about 50% of patients surviving at 10 years from diagnosis [1,2,3]. Indeed, novel drugs, such as immunomodulatory agents (IMiDs) and proteasome inhibitors (PIs), significantly increased the life expectancy of MM patients in the last decades. The standard treatment approach for transplant-eligible newly diagnosed MM (NDMM) patients includes a novel agent-based induction followed by high-dose chemotherapy and autologous stem-cell transplantation (ASCT). In this patient population, post-ASCT maintenance therapy with lenalidomide until progressive disease (PD) has been approved by both the European Medicines Agency (EMA) and the Food and Drug Administration (FDA). A recently published meta-analysis, including data from three randomized trials, showed a significant benefit in terms of progression-free survival (PFS) and OS for patients receiving lenalidomide maintenance after ASCT compared to the observation or placebo [4]. In the transplant-ineligible setting, continuous therapy with novel agents is one of the current standard approaches. Among the available choices, lenalidomide plus dexamethasone (Rd) until PD and daratumumab plus bortezomib-melphalan-prednisone (VMP) followed by daratumumab maintenance until PD improved PFS, as compared to fixed-duration therapy [5,6,7]. In transplant-ineligible patients, a randomized trial showed that, after melphalan–prednisone–lenalidomide (MPR) induction, lenalidomide maintenance until PD was associated with a better PFS compared to no maintenance, although no OS advantage was observed [8].

Steroids have been used in the past as maintenance therapy and a randomized trial showed a significant PFS benefit in patients receiving maintenance with pharmacological vs. physiological doses of prednisone [9]. In another study, dexamethasone maintenance resulted in a significant PFS advantage, as compared to observations in elderly NDMM patients after induction with melphalan and steroids [10]. Prednisone has also been combined with thalidomide maintenance, showing a PFS benefit and conflicting results in terms of OS [11,12].

Currently, there are no clear data on the potential advantage of continuous maintenance until PD compared to stopping after a prolonged but fixed duration of therapy (i.e., 18 or 24 months). In two of the three trials included in the aforementioned meta-analysis of post-ASCT lenalidomide maintenance, treatment was continued until PD; nevertheless, in the IFM2005-02 trial by the Intergroupe Francophone du Myélome (IFM) a subset of patients stopped maintenance before PD due to the increased concern about second primary malignancies (SPMs) related to lenalidomide [4,13].

The primary aim of this individual patient data meta-analysis was to evaluate if adding prednisone (a long-established and affordable drug, easily available all around the world) at a dose of 25/50 mg every other day (eod) to lenalidomide maintenance could be effective and safe. The secondary aim was to analyze the benefit of treatment with lenalidomide until PD vs. up to two years on long-term outcomes and on outcomes after the first relapse.

## 2. Methods

Data from two phase III multicenter international studies (RV-MM-EMN-441 and EMN01) were pooled together and analyzed. In the RV-MM-EMN-441 trial, transplant-eligible NDMM patients received an induction therapy with 4 cycles of lenalidomide and dexamethasone (Rd) followed by chemotherapy with cyclophosphamide and stem-cell mobilization and collection. The consolidation regimen consisted of 6 cycles of cyclophosphamide, lenalidomide and dexamethasone (CRD) vs. tandem high-dose melphalan plus ASCT. After consolidation, patients received maintenance therapy with lenalidomide and prednisone (RP) vs. lenalidomide alone (R) [14]. In the EMN01 trial, transplant-ineligible NDMM patients were randomized to receive induction with 9 cycles of Rd vs. MPR vs. cyclophosphamide, prednisone and lenalidomide (CPR). After induction, patients received maintenance therapy with R or RP [15]. In both studies, lenalidomide-based maintenance was administered until PD or development of unacceptable toxicity. Lenalidomide was given at the dose of 10 mg on days 1 to 21 of 28-day cycles in both trials; prednisone was administered at the dose of 50 mg eod in RV-MM-EMN-441 and of 25 mg eod in EMN01. The two studies were respectively approved by the institutional review boards at each of the participating centers and are registered at ClinicalTrials.gov (numbers NCT01091831 and NCT01093196). All patients provided written informed consent before entering the source trials. The work described has been carried out in accordance with The Code of Ethics of the World Medical Association (Declaration of Helsinki) for experiments involving humans.

The primary aim of our analysis was to evaluate the efficacy of adding prednisone to lenalidomide maintenance. We therefore analyzed PFS, progression-free survival 2 (PFS2) and OS in patients receiving maintenance with RP vs. R. Secondary endpoints were time to next treatment (TTNT) and the toxicity profile of patients treated with RP vs. R. The comparative analysis included all patients eligible for maintenance and the starting time was the date of clinical assessment for maintenance eligibility after CRD or MEL200 in the RV-MM-EMN-441 trial, and after Rd/MPR/CRP in the EMN01 trial [16].

A further secondary aim was to evaluate the impact of duration of lenalidomide therapy on long-term outcome (OS from start of maintenance) and on outcome after the first relapse (second PFS and OS from the relapse). We classified patients into two groups: the long-term maintenance group included patients continuing maintenance for more than 2 years, and the short-term maintenance group included patients stopping maintenance before 2 years. Since early progression is a negative prognostic feature per se, we excluded from this analysis patients who stopped maintenance before two years due to PD. We compared baseline features and response during maintenance in the two groups to evaluate any differences that could affect outcome.

In a subsequent analysis, in order to also evaluate the impact on outcome of a shorter duration of maintenance (1 year), we compared OS, PFS from relapse and OS from relapse between patients stopping maintenance before one year, patients continuing maintenance for more than one year but for less than two years, and patients continuing maintenance for more than two years.

Endpoint definitions and further statistical considerations can be found in the Supplementary Materials.

## 3. Results

### 3.1. Patients

**Figure 1:**
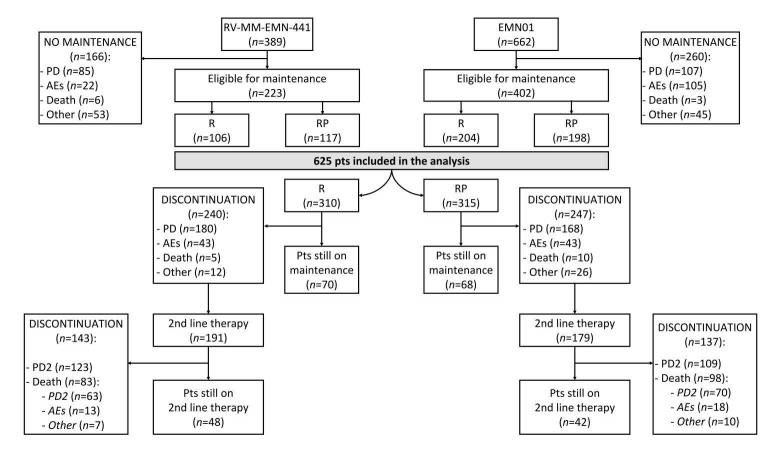
Study flow. Abbreviations: *n*, number of patients, R, lenalidomide; RP, lenalidomide, prednisone; AEs, adverse events; PD, progressive disease; pts, patients; 2nd, second.

A total of 1051 patients were enrolled in the two trials; 625 were eligible for maintenance and therefore included in this analysis. A total of 315 patients received maintenance with RP and 310 with R (Figure 1).

Main reasons for not starting maintenance were disease progression (192 patients, 18%) and adverse events (AEs, 127 patients, 12%). A higher proportion of patients with Revised International Staging System (R-ISS) stage I disease (23% vs. 17%, *p* = 0.03), a higher rate of very good partial response/partial response (VGPR/PR, 81% vs. 72%, *p* < 0.001) and a lower rate of stable disease (SD, 12% vs. 23%, *p* < 0.001) were observed in the maintenance population, as compared with the overall population of the two trials. Main patient characteristics and disease response before maintenance were well balanced between patients receiving RP vs. R maintenance (Table 1).

Median age at diagnosis was 69 years for the RP arm and 70 years for the R arm. Fifty-eight patients (19%) in the RP arm and 60 patients (18%) in the R arm had International Staging System (ISS) stage III. High-risk cytogenetic features (t(4;14) and/or t(14;16) and/or del(17p)) were present in 59 (24%) and 55 patients (23%) in the RP and R arms, respectively. Seventeen patients (6%) in the RP arm and 15 patients (6%) in the R arm had R-ISS stage III. Two hundred and seventy-nine (89%) patients in the RP arm and 269 (87%) patients in the R arm achieved at least a partial response (≥PR) before starting maintenance. The median duration of maintenance was 27 months in the RP arm and 22 months in the R arm. At data cut-off, after a median follow-up of 58 months, 70 patients (23%) in the R arm and 68 patients (22%) in the RP arm were still receiving lenalidomide-based maintenance. Main reasons for the discontinuation of the maintenance treatment were PD (58% vs. 53% of patients in R and RP groups, respectively) and AEs (12% in both groups). In the overall population, 370 patients (59%) started a second-line therapy. Most patients (*n* = 286, 77%) received a PI-based therapy; 27 patients (7%) received ASCT as a second-line therapy (Table S1).

### 3.2. PFS, TTNT, PFS2 and OS Analysis of RP vs. R Maintenance

**Figure 2:**
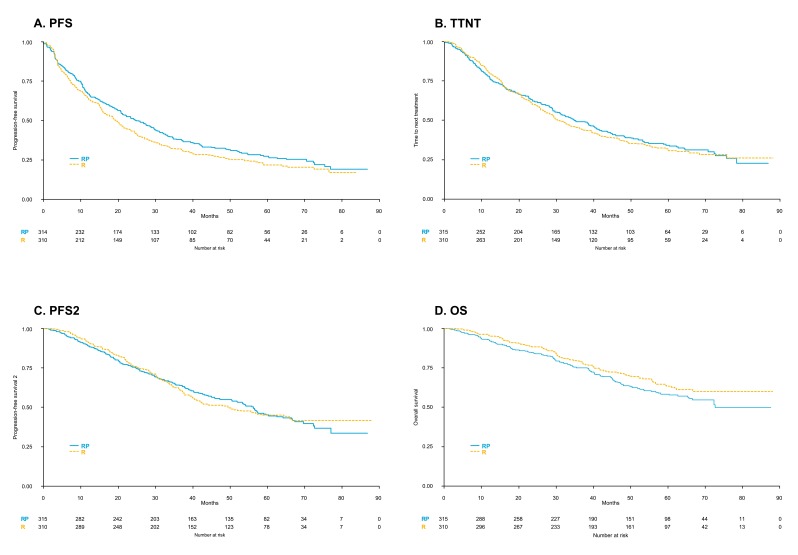
Outcome in patients receiving lenalidomide-prednisone (RP) vs. lenalidomide (R) alone: (**A**) progression-free survival (PFS); (**B**) time to next treatment (TTNT); (**C**) progression-free survival 2 (PFS2) and (**D**) overall survival (OS).

A modest benefit in terms of PFS was observed in the RP vs. R arms (median PFS 25 months vs. 19 months, hazard ratio (HR) = 0.86, 95% confidence interval (CI) = 0.72–1.03, *p* = 0.08; Figure 2A), although the difference was not statistically significant. Median TTNT was about 10 months longer than median PFS in both arms, with no significant differences between the RP and R groups (median 35 vs. 30 months, HR = 0.96, 95% CI = 0.79–1.16, *p* = 0.63; Figure 2B). No significant differences in PFS2 (median 56 vs. 49 months, HR = 1.00, 95% CI = 0.81–1.25, *p* = 0.98; Figure 2C) and OS (5 years: 58% vs. 63%, HR = 1.26, 95%, CI = 0.96–1.64, *p* = 0.08; Figure 2D) were noticed.

The subgroup analysis showed no benefit of RP vs. R in terms of PFS, TTNT, PFS2 and OS in any of the subgroups analyzed according to age, R-ISS stage and disease response before starting maintenance (Figure S1A–D).

In multivariate Cox regression analysis, including baseline features and response before maintenance, R-ISS stage resulted to be the main independent predictor of PFS, TTNT, PFS2 and OS (Table 2).

### 3.3. Safety Profile of RP vs. R Maintenance

Both RP and R were well tolerated. All-grade toxicities occurring in >10% of patients and Grade 3–4 toxicities are summarized for each group in Table 3.

No significant differences between the two groups were found in terms of hematologic AEs, with the exception of neutropenia (grade ≥ 3: 58 (19%) vs. 27 patients (9%), *p* < 0.001). The higher rate of neutropenia in the R arm did not lead to an increased infection rate (grade ≥ 3: 5 (2%) vs. 10 patients (3%), *p* = 0.418). The most frequent non-hematologic toxicity other than infections was diarrhea (any grade: 35 (11%) vs. 21 patients (7%) in the R vs. RP arm, respectively, *p* = 0.049; grade ≥ 3: 0 vs. 3 patients (1%) in the R vs. RP arm, *p* = 0.248). The incidence of SPMs during maintenance was low in both groups (13 (4%) vs. 14 patients (4%) in the R vs. RP arms, *p* = 1.000) and mainly represented by skin carcinomas. In detail, in the R group 7 patients developed a skin carcinoma, 2 an adenocarcinoma, 1 a meningioma, 1 a glioblastoma, 1 a renal cancer and 1 patient developed a myelodysplastic syndrome. In the RP group, 9 patients developed a skin carcinoma, 3 a urothelial carcinoma, 1 patient an adenocarcinoma and 1 patient developed breast cancer. No deaths related to AEs occurred during maintenance in the R group; in the RP group 3 deaths were recorded (septic shock *n* = 1; gastrointestinal hemorrhage *n* = 1; and respiratory failure *n* = 1). Lenalidomide dose reductions due to AEs were more frequent in the R group than in the RP group (51 (16%) vs. 27 patients (8%), *p* = 0.003) with no significant differences between the two trials. The median cumulative dose percentage of lenalidomide was 100% in both groups. In the R group, the main toxicities leading to lenalidomide dose reductions were hematologic toxicities (20 patients), diarrhea (8 patients) and rash (7 patients). In the RP group, the main reasons for lenalidomide dose reductions were hematologic toxicities (5 patients), diarrhea (5 patients) and peripheral neuropathy (5 patients). In the RP arm, prednisone dose reductions due to AEs were necessary for 54 patients (17%). Prednisone dose reductions due to AEs were more frequent in patients receiving 50 mg eod (RV-MM-EMN-441, 42 patients (36%)) than in those receiving 25 mg eod (EMN01, 12 patients (6%); *p* < 0.001). The main reasons for prednisone dose reductions were metabolic intolerance (weight gain and/or hyperglycemia) for 11 patients and psychiatric alterations (such as agitation and/or insomnia) for 9 patients.

Forty-three patients (14%) discontinued lenalidomide due to an AE in both R and RP groups. The most frequent reasons for lenalidomide discontinuation were SPMs (9 and 6 patients, respectively), infections (4 and 3 patients), hematologic toxicities (3 and 4 patients) and rash (3 and 2 patients). In the RP group, prednisone discontinuation due to an AE occurred in 53 patients (17%), with no significant differences between the two trials (RV-MM-EMN-441: 19 patients (16%) vs. EMN01: 34 patients (17%); *p* = 0.877). The most frequent toxicities leading to prednisone discontinuation were infections and mood alterations (4 patients each). The overall feasibility of maintenance was not affected by prior induction treatment, with in particular no negative impact of more intensive pre-maintenance therapy on the overall duration of maintenance treatment (Table S3).

### 3.4. Impact of Duration of Maintenance on Outcome

We compared the outcome of patients continuing maintenance for more than 2 years (long-term maintenance group) and patients stopping maintenance before 2 years (short-term maintenance group). The patients who discontinued maintenance before 2 years due to PD were excluded to avoid a negative selection bias; AEs were the main reason for early maintenance discontinuation in this subgroup of patients. Patients in the short-term group were older than those in the long-term group (48 (80%) vs. 188 (60%) patients ≥65 years old, respectively; *p* = 0.003). A significantly higher proportion of patients in the long-term maintenance group improved response during maintenance (139 (44%) vs. 14 (23%); *p* = 0.002), resulting in a significantly higher number of patients achieving at least a very good PR (≥VGPR, 217 (69%) vs. 21 (35%) patients, respectively, *p* < 0.001; Table S2).

**Figure 3:**
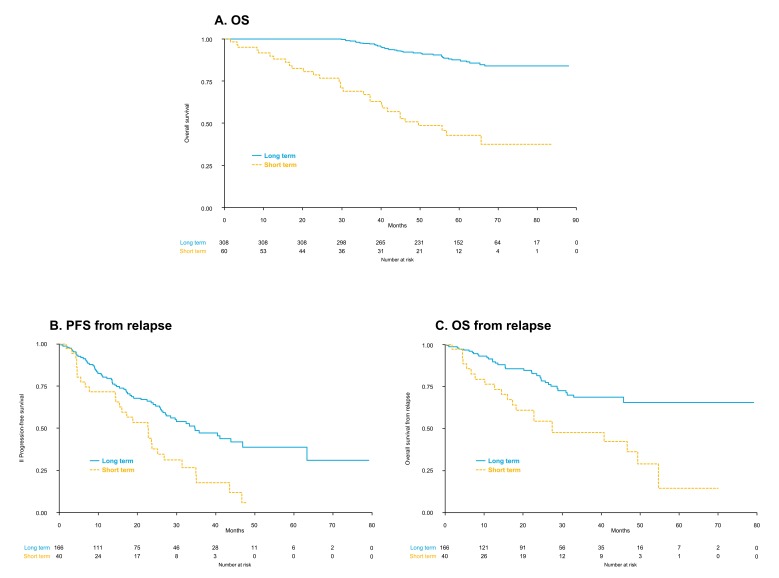
Outcome in patients continuing maintenance for more than 2 years (long-term maintenance) vs. stopping before 2 years (short-term maintenance): (**A**) overall survival (OS); (**B**) progression-free survival (PFS) from relapse; and (**C**) overall survival (OS) from relapse.

A significant advantage in OS was observed for patients in the long-term maintenance group, as compared to patients in the short-term group (median not reached vs. 49.6 months, *p* < 0.001; Figure 3A). In a multivariate analysis (which included age, R-ISS stage, treatment with R/RP and response), the duration of maintenance was the main predictor of OS (HR = 0.17; 95% CI = 0.10–0.29; *p* < 0.001).

Patients who continued maintenance for more than two years showed significantly longer PFS from relapse (median 34.7 vs. 23.6 months, *p* = 0.004; Figure 3B) and OS from relapse (median not reached vs. 40.7 months, *p* = 0.002; Figure 3C).

The patients who continued maintenance therapy for more than 2 years showed a significant advantage in terms of OS and PFS from relapse, also when compared to patients who continued maintenance for more than 1 year but for less than 2 years (median OS: NR in both groups, HR = 0.27, 95% CI = 0.13–0.56, *p* < 0.001; median PFS from relapse: 34.7 vs. 22.8 months, HR = 0.45, 95% CI = 0.22–0.89, *p* = 0.023). A trend toward better OS from relapse was also observed (median OS from relapse NR vs. 40.7 months, HR = 0.5, 95% CI = 0.21–1.2, *p* = 0.120; results are shown in Figure S2 in the Supplementary Materials).

## 4. Discussion

The results of this pooled analysis showed that the addition of prednisone at a dose of 25 or 50 mg eod to lenalidomide maintenance did not induce any significant advantage. Despite a trend toward better PFS in the RP-treated vs. the R-treated group, no significant TTNT, PFS2 and OS benefits were found. Regarding PFS according to prior induction administered, only a trend towards a better PFS in the MEL200 arm of the RV-MM-EMN-441 study was observed, but with no significant difference. Unfortunately, both trials included in our analysis were not powered to detect differences between RP or R in each pre-maintenance induction group [14,15,17,18]. In a previous study, maintenance treatment with a therapeutic dose of steroids (prednisone: 50 mg eod) showed a benefit in terms of PFS (median, 14 vs. 5 months; *p* = 0.003) and OS (median, 37 vs. 26 months; *p* = 0.05) compared to a lower physiological dose (prednisone: 10 mg eod) [9]. The lack of benefit observed in our study may partly rely on the fact that prednisone at the dose of 50 mg eod was scarcely tolerated in the long term, with more than one-third of patients requiring dose reductions. Our results are in line with those of the recently presented RV-MM-PI-0752 trial, which did not show any PFS/OS benefit of continuing dexamethasone after 9 Rd cycles, as compared with treatment with lenalidomide alone [19].

At the current follow-up of approximatively 5 years, the median OS was 72 months in RP-treated and not reached in R-treated patients. Lenalidomide maintenance was well tolerated in the overall population. The lower incidence of neutropenia observed in the RP group can be explained by the compensatory effect of steroid-induced leukocytosis. Indeed, steroids induce the demargination of neutrophils from endothelial cells and inhibit the migrations of polymorphonuclear cells (PMNs) into tissues [20,21]. Despite a lower rate of lenalidomide dose reductions in the RP group (related to a lower rate of neutropenia), lenalidomide discontinuation rates due to AEs where similar in the 2 arms. It could be expected that patients receiving higher intensity inductions would experience a higher rate of maintenance discontinuation due to AEs, likely due to higher toxicity from previous therapy. Nevertheless, we did not observe this effect. In particular, in the RV-MM-EMN-441 study, the feasibility of maintenance therapy in patients treated with MEL200 vs. CRD was comparable [14]. Duration of maintenance is affected both by efficacy and tolerability. In the RV-MM-EMN-441 study, the median durations of maintenance were 28.9 months in the RP group and 25.3 months in the R group, respectively. These durations were longer than those observed in the EMN01 trial (23 months in the RP group vs. 20.5 months in the R group [15,18]), although treatment administered in the RV-MM-EMN-441 trial was more intense. This was likely because younger patients can tolerate better maintenance, but also because more intensive pre-maintenance treatments can induce higher cytoreduction and disease control, as shown by the longest duration of maintenance reported in patients treated with MEL200.

The SPM rate (4%) observed in our analysis is similar to that reported in the Myeloma XI trial (3.8%), and lower than those observed in the CALGB (7.8%), IFM 2005-02 (10%) and MM-015 (7.9 %) studies [8,13,22,23]. A possible explanation is the lower proportion of patients (39%) in our analysis who received melphalan, as compared to other trials. In fact, it has been reported that the SPM risk might be increased by the administration of lenalidomide with or after melphalan [24].

The RP vs. R comparison has some limitations. First, the intensities of pre-maintenance therapies in the two trials included in this metanalysis were different from each other, and consolidation was not included in the EMN01 trial. This is an unavoidable consequence of the fact that the two trials enrolled different patient populations. Second, the doses of prednisone in the RP arms of the two studies were different. Nevertheless, the multivariate regression model, which was adjusted for trial effect and therefore accounted for the trial differences, confirmed no benefit of RP.

Finally, the selected doses of prednisone could be suboptimal, in particular the 25 mg eod dose. It should be, however, noted that 25 mg eod correspond to a weekly dose of 16 mg of dexamethasone, which is only slightly inferior to the dose of 20 mg of dexamethasone recommended in many trials for elderly patients.

The optimal duration of maintenance, and in particular the need for continuous treatment until PD vs. a prolonged but fixed duration of therapy (e.g., 1 or 2 years), still remains an open issue. Most trials conducted so far have explored lenalidomide maintenance until PD [8,13,14,15,22,23,25]. Unfortunately, there are no available results coming from randomized studies prospectively evaluating the optimal duration of maintenance treatment. Nevertheless, this is a very important issue, both from a patient perspective (in order to avoid unnecessary toxicities) and for pharma-economic reasons. We tried to evaluate the possible impact of different durations of maintenance on long-term outcome. We chose the 2-year cut-off since it was the median duration of maintenance in the two trials included in our analysis [14,15], as well as in other published reports [5,22]. These data support 2 years as a reasonably tolerable minimum treatment period.

Patients in the long-term group had better OS (median NR vs. 49.6 months; *p* <0.001), as well as better PFS and OS from relapse (median PFS from relapse: 34.7 vs. 23.6 months, *p* < 0.001; median OS from relapse: NR vs. 40.7 months, *p* = 0.002). The main limitation of this analysis is that, not being a randomized comparison, there are differences between the populations of patients in the two groups. In the short-term group, a higher proportion of patients were >65 years old. This finding could contribute to the inferior outcome observed. In the short-term group, we excluded patients who discontinued maintenance before 2 years due to PD in order to avoid a negative selection bias, thus including in this group only those patients sensitive to the drug itself but unable to receive prolonged therapy. Of note, a significantly higher proportion of patients who were able to continue therapy for more than 2 years (long term-maintenance group) improved their response, and this can explain the better outcome. Minimal residual disease (MRD) was not systematically performed in the two trials included in our analysis, but recent data from the EMN02 study showed that also the MRD rate improves during maintenance, with 50% of patients who are MRD positive before maintenance who turn MRD negative within the first 2 years of maintenance [26]. The impact of MRD-negative status on both PFS and OS is well known [27]. Our findings are similar to those reported in another retrospective analysis, in which a duration of maintenance >2 years vs. ≤2 years was associated with longer OS (HR = 0.09, 95% CI = 0.03–0.26, *p* < 0.001) [28]. Of note, in our analysis, patients who continued maintenance for more than 2 years showed better OS and PFS from relapse, as compared to patients who continued maintenance for more than 1 year but for less than 2 years, further supporting the idea that prolonged maintenance treatment for at least 2 years can be beneficial.

In conclusion, our study showed that the combination of lenalidomide and prednisone at 25/50 mg eod did not improve outcome compared with lenalidomide alone as a maintenance strategy. Steroids are pivotal in the treatment landscape for multiple myeloma, since they are easily available and effective agents that show synergistic activity in combination with other drugs. Yet, if a doublet is needed as maintenance, which patients may benefit from it, which steroid dose could be optimal, and which agent could be the best partner of lenalidomide, remain open questions. The potential benefit of steroids in association with other agents as maintenance strategy is also still unknown. Ongoing clinical trials are evaluating the role of other novel agents and combinations, including PIs [29,30,31], monoclonal antibodies [32,33] and histone deacetylase inhibitors [34]. The optimal duration of therapy is also still an important issue. In the absence of prospective randomized comparisons, and despite the already-mentioned limitations of our analysis, the available data support prolonged treatment for at least 2 years [35]. Nevertheless, in the era of MRD, studies assessing MRD-driven strategies are needed to further optimize patient management.

## Figures and Tables

**Table 1:** Main patient characteristics

Characteristics	All Patients	Patients Starting Maintenance	R	RP
	(*n* = 1051)	(*n* = 625)	(*n* = 310)	(*n* = 315)
**PROTOCOL**				
RV-MM-EMN-441	389 (37%)	223 (36%)	106 (34%)	117 (37%)
EMN01	662 (63%)	402 (64%)	204 (66%)	198 (63%)
**INDUCTION**				
MEL200	195 (19%)	117 (19%)	57 (18%)	60 (19%)
CRD	194 (18%)	106 (17%)	49 (16%)	57 (18%)
MPR	218 (21%)	126 (20%)	65 (21%)	61 (19%)
CRP	222 (21%)	143 (23%)	76 (25%)	67 (22%)
Rd	222 (21%)	133 (21%)	63 (20%)	70 (22%)
**AGE**				
median (range)	70 (18–111)	69 (59–74)	70 (60–75)	69 (59–74)
>75 years old	230 (22%)	125 (20%)	61 (20%)	64 (20%)
71–75 years old	243 (23%)	158 (25%)	88 (28%)	70 (22%)
61–70 years old	321 (31%)	175 (28%)	78 (25%)	97 (31%)
≤60 years old	257 (24%)	167 (27%)	83 (27%)	84 (27%)
**GENDER**				
Female	531 (51%)	327 (52%)	175 (56%)	152 (48%)
Male	518 (49%)	298 (48%)	135 (44%)	163 (52%)
**R-ISS STAGE °**				
I	153 (17%)	115 (23%)	51 (21%)	64 (24%)
II	661 (74%)	362 (71%)	181 (73%)	181 (70%)
III	73 (8%)	31 (6%)	15 (6%)	17 (6%)
NA	164	116	63	53
**CHROMOSOMAL ABNORMALITIES (FISH)°**				
Standard risk	599 (73%)	369 (76%)	180 (77%)	189 (76%)
High risk	216 (27%)	114 (24%)	55 (23%)	59 (24%)
NA	236	142	75	67
**BEST RESPONSE BEFORE MAINTENANCE**				
sCr/CR	49 (5%)	44 (7%)	20 (6%)	24 (8%)
VGPR/PR	719 (72%)	504 (81%)	249 (80%)	255 (81%)
SD	228 (23%)	77 (12%)	41 (13%)	36 (11%)

* High-risk is defined by the presence of at least one among del(17p), t(4;14) and/or t(14;16); ° % calculated on the number of patients whose data were available. R, lenalidomide; RP, lenalidomide-prednisone; R-ISS, Revised International Staging System; MEL200, melphalan at 20 mg/mq; CRD, cyclophosphamide-lenalidomide-dexamethasone; MPR, melphalan-prednisone-lenalidomide; CPR, cyclophosphamide-prednisone-lenalidomide; Rd, lenalidomide-dexamethasone; FISH, fluorescent in situ hybridization; NA, not available; sCR, stringent complete response; CR, complete response; VGPR, very good partial response; PR, partial response; SD, stable disease; *n*, number.

**Table 2:** Multivariate Cox regression analysis of main baseline predictors of outcome in patients receiving lenalidomide-based maintenance

Cox Model	HR	95% CI	*p* Value
PFS			
**Stage**			
R-ISS II vs. I	1.90	1.47–2.46	<0.001
R-ISS III vs. I	2.54	1.65–3.93	<0.001
**TTNT**			
**Stage**			
R-ISS II vs. I	2.06	1.55–2.74	<0.001
R-ISS III vs. I	2.98	1.88–4.71	<0.001
**PFS2**			
**Stage**			
R-ISS II vs. I	2.55	1.79–3.63	<0.001
R-ISS III vs. I	4.03	2.41–6.73	<0.001
**OS**			
**Stage**			
R-ISS II vs I	2.33	1.52–3.57	<0.001
R-ISS III vs I	4.37	2.45–7.80	<0.001

PFS, progression-free survival; TTNT, time to next treatment; PFS, progression-free survival 2; OS, overall survival; HR, hazard ratio; CI, confidence interval; R-ISS, Revised International Staging System.

**Table 3:** Main treatment-related adverse events during maintenance. Number (%) of patients with at least one adverse event

	Any Grade			Grade 3–4		
Adverse Events	R (*n* = 310)	RP ((*n* = 315)	*p* Value	R ((*n* = 310)	RP ((*n* = 315)	*p* Value
**HEMATOLOGIC**	168 (54%)	125 (40%)	0.003	62 (20%)	36 (11%)	0.004
** Anemia**	65 (21%)	63 (20%)	-	4 (1%)	5 (2%)	-
** Neutropenia**	108 (35%)	67 (21%)	<0.001	58 (19%)	27 (9%)	<0.001
** Thrombocytopenia**	45 (15%)	39 (12%)	-	5 (2%)	6 (2%)	-
**NON-HEMATOLOGIC**	142 (46%)	172 (55%)	0.03	39 (13%)	54 (17%)	-
** Infection**	30 (10%)	35 (11%)	-	5 (2%)	9 (3%)	-
*Pneumonia*	5 (2%)	6 (2%)	-	2 (1%)	1 (1%)	-
*Bronchitis*	6 (2%)	5 (2%)	-	0	1 (1%)	-
*VZV infection*	2 (1%)	4 (1%)	-	0	1 (1%)	-
** Fatigue**	11 (4%)	20 (6%)	-	2 (1%)	1 (1%)	
** Gastrointestinal**	53 (17%)	37 (12%)	-	0	5 (2%)	
*Diarrhea*	35 (11%)	21 (7%)	-	0	3 (1%)	-
*Constipation*	4 (1%)	5 (2%)	-	0	0	-
*Nausea*	2 (1%)	3 (1%)	-	0	0	
** Peripheral neuropathy**						
*PNP and paresthesia*	5 (2%)	17 (5%)	0.01	1 (1%)	4 (1%)	-
*Cramps*	3 (1%)	6 (2%)	-	0	1 (1%)	-
Central nervous system *	5 (2%)	16 (5%)	0.02	2 (1%)	4 (1%)	-
** VTE**						
*Deep vein thrombosis*	3 (1%)	8 (3%)	-	3 (1%)	6 (2%)	-
*Pulmonary embolism*	1 (1%)	3 (1%)	-	1 (1%)	3 (1%)	-
** Dermatological**	19 (6%)	11 (3%)	-	3 (1%)	4 (1%)	-
*Rash*	12 (4%)	7 (2%)	-	3 (1%)	3 (1%)	-

* Central nervous system adverse events mainly consisting of mood alterations (such as agitation or irritability) related to prednisone. The *p* values were included when significant. *n*, number; R, lenalidomide; RP, lenalidomide-prednisone; VZV, Varicella zoster virus; PNP, peripheral neuropathy; VTE, venous thromboembolism.

## Supplementary Materials

The following are available online at https://www.mdpi.com/2072-6694/11/11/1735/s1: Additional Methods: Endpoints, Statistical analysis, Table S1: Second-line therapies, Table S2: Patient characteristics and best response achieved during maintenance in patients receiving short vs. long-term maintenance duration, Table S3: Median duration of maintenance according to the different induction therapies administered, Figure S1: Hazard ratios (HRs) by subgroups for progression-free survival (PFS), time to next treatment (TTNT), PFS2 and overall survival (OS) in the lenalidomide-prednisone (RP) vs lenalidomide (R) comparison, Figure S2: Comparison between patients continuing maintenance for more than two years (long term) vs more than 1 year but less than 2 years (medium term) vs less than 1 year (short term) in terms of OS, PFS from relapse, and OS from relapse.
